# Biomechanical application of finite elements in the orthopedics of stiff clubfoot

**DOI:** 10.1186/s12891-022-06092-0

**Published:** 2022-12-21

**Authors:** Wei Liu, Fei Li, Haiyang He, Aihelamu Teraili, Xue Wang, Paerhati Wahapu, Chengwei Wang

**Affiliations:** 1 The Affiliated Tumor Hospital of Xinjiang Medical University, Urumqi, Xinjiang 830000 People’s Republic of China; 2 The Sixth Affiliated Hospital of Xinjiang Medical University, Urumqi, Xinjiang 830000 People’s Republic of China

**Keywords:** Finite element analysis, Triple arthrodesis, Hindfoot alignment angle, Biomechanics, Arthritis

## Abstract

**Background:**

The purpose of this study was to evaluate the effect of varying the different correction angles of hindfoot osteotomy orthosis on the biomechanical changes of the adjacent joints after triple arthrodesis in adult patients with stiff clubfoot to determine the optimal hindfoot correction angle and provide a biomechanical basis for the correction of hindfoot deformity in patients with stiff clubfoot.

**Methods:**

A 26-year-old male patient with a stiff left clubfoot was selected for the study, and his ankle and foot were scanned using dual-source computed tomography. A three-dimensional finite element model of the ankle was established, and after the validity of the model was verified by plantar pressure experiments, triple arthrodesis was simulated to analyze the biomechanical changes of the adjacent joints under the same load with “3°” of posterior varus, “0°” of a neutral position and “3°, 6°, 9°” of valgus as the correction angles.

**Results:**

The peak plantar pressure calculated by the finite element model of the clubfoot was in good agreement with the actual plantar pressure measurements, with an error of less than 1%. In triple arthrodesis, the peak von Mises stress in the adjacent articular cartilage was significantly different and less than the preoperative stress when the corrected angle of the hindfoot was valgus “6°”. In comparison, the peak von Mises stress in the adjacent articular cartilage was not significantly different in varus “3°”, neutral “0°”, valgus “3°” and valgus “9°” compared with the preoperative stress.

**Conclusion:**

The results of this study showed that different angles of hindfoot correction in triple arthrodesis did not increase the peak von Mises stress in the adjacent joints, which may not lead to the development of arthritis in the adjacent joint, and a hindfoot correction angle of “6°” of valgus significantly reduced the peak von Mises stress in the adjacent joints after triple arthrodesis.

## Introduction

Stiff clubfoot is a complex three-dimensional deformity with deformities associated with “talocalcaneal (ST), talonavicular (TN), and calcaneocuboid (CC) joints. Triple arthrodesis (TA) is a safe and reliable surgical procedure to stabilize the foot, correct the foot deformity and stop further development of the foot deformity; patient satisfaction after surgery is also high, and TA has now become a standard surgical procedure for the correction of stiff clubfoot [1, 2], intending to correct all deformity features and obtain a painless metatarsal foot. Nevertheless, degenerative lesions of the adjacent joints are often seen after TA of stiff clubfoot [3], leading to pain in the adjacent joints of the affected limb and even the possibility of reoperation. Some studies have shown that correct alignment of the midfoot with the forefoot after TA does not lead to the development or progression of adjacent joint arthritis [4]. Nevertheless, the proper alignment of the hindfoot was not included as a parameter in the study. Some studies have reported that the development or aggravation of adjacent joint arthritis after TA is associated with the patient’s preexisting adjacent joint arthritis and/or persistent TA. TA does not lead to the development of osteoarthritis in the adjacent joints or aggravate the progression of osteoarthritis in the adjacent joints in the presence of a correctly aligned hindfoot [5]. Nevertheless, this study lost a significant number of patients to follow-up. The clinical results of these studies were obtained through long-term follow-up of the patients, but the mechanism of how hindfoot alignment after TA affects the adjacent joints was not mentioned.

It has been proposed that an adequately aligned hindfoot is a neutral “0°” hindfoot obtained by translating the calcaneus axis parallel to the tibial axis and then bringing the calcaneus axis in line with the tibial axis [6]; however, no clinical validation or relevant biomechanical studies have been performed. BUCK et al. [7] considered correct hindfoot alignment as valgus “0° to 10°” and considered any angle of hindfoot varus to be an abnormal foot; some studies also reported normal human hindfoot alignment angle as valgus “2° to 5°” using radiological methods " and valgus “5.61° to 6.50°” as measured by clinical examination methods [8], however, this result was included in a smaller sample size. The more accepted angle of correct hindfoot alignment is “0° to 5°” of valgus obtained on radiographs in the Saltzman position [9]. No trials or reports have evaluated the effects of normal hindfoot alignment and different hindfoot correction angles on the adjacent joints postoperatively after triple arthrodesis for stiff clubfoot.

The biomechanical changes in the adjacent joints after triple arthrodesis in patients with stiff clubfoot are significant in guiding the selection of clinical treatment plans as the orthopedic angle of the stiff clubfoot changes. Therefore, we developed a three-dimensional finite element model of the stiff clubfoot to predict the local biomechanical distribution of the adjacent joints after triple arthrodesis and investigated the results of the biomechanical changes of the adjacent joints at different orthopedic angles of the stiff clubfoot hindfoot to determine the optimal hindfoot orthopedic angle for triple arthrodesis.

## Materials and methods

### Geometry design

To evaluate the biomechanical trends of the adjacent joints at different correction angles of the hindfoot during triple arthrodesis of the stiff clubfoot, we obtained a finite element model of the affected limb of a patient with stiff clubfoot, including the whole foot and its tibiofibular, based on CT images of the affected limb of a 25-year-old male volunteer (weight = 50 kg, height = 1.65 m) with left-sided stiff clubfoot. The study was conducted according to the principles of the Declaration of Helsinki, and this volunteer gave written informed consent to participate in our research. He had no soft tissue, joint, or skeletal lesions other than those of the affected limb, and his preoperative radiographs did not suggest arthritis of the adjacent joints. We first used Siemens dual-source CT to scan the lower extremities of this volunteer (voltage 120 kV, current intensity 240 mA, scan slice thickness 0. 600 mm) and obtained a total of 780 DICOM 2D tomographic images. The 3D model of the stiff clubfoot, including the tibia, fibula, and 30 bones of the foot (tibia, fibula, talus, calcaneus, navicular bone, cuboid, three cuneiform bones, five metatarsal bones, 14 toes, and two seed bones) was saved in STL format. The STL files of the above 30 bones were imported into the reverse engineering software Geomagic Wrap. After removing the nail, noise reduction, smoothing, and removing the pin, noise reduction, and smooth and delicate surface operations, the nonuniform rational B-splines (NURBS) surface models of the above 30 skeletons were obtained. The models were imported into SolidWorks in STP format. According to the patient’s CT, we took a thickness of 2 mm as the cortical bone and obtained 60 pieces of cortical bone and cancellous bone models. Frictionless face-to-face contact was used to represent the relative joint motion between the articular cartilage layers to achieve frictionless sliding between the bones. One hundred two-foot ligaments were modeled using rod units that can only be stretched but not compressed based on the anatomical information on the digital anatomical platform and the human atlas. These ligaments were manually positioned and added to the model according to the relevant anatomical landmarks, similar to previous studies [10–13]. Sixty bone models, 37 cartilage models, and 102 ligament models were obtained. All bones, cartilage, and ligaments were assembled into the same model.

### The assignment of material properties

**Fig. 1:**
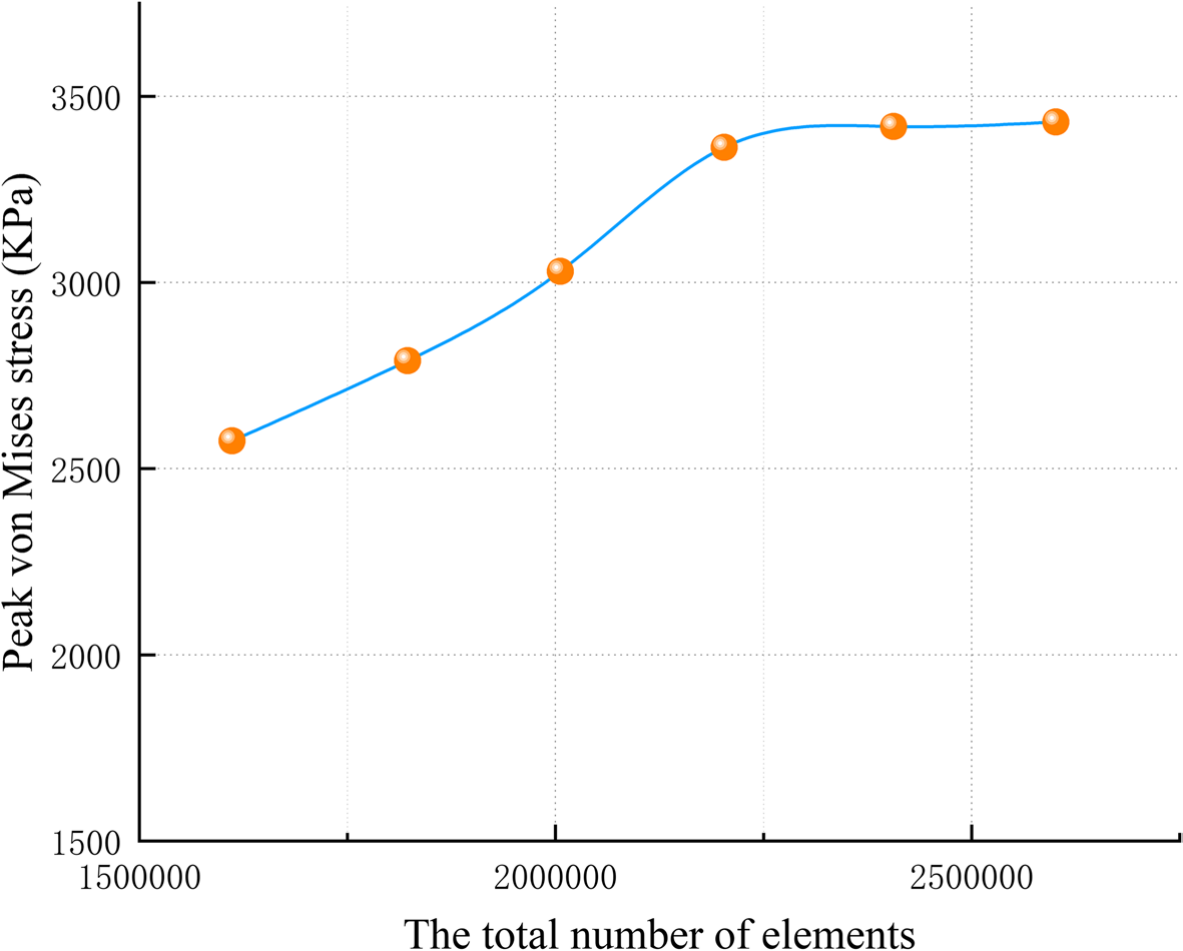
Convergence tests results in terms of the peak von Mises stress

We created tetrahedral mesh cells on the stiff clubfoot model by Hypermesh software with a mesh size of 2 mm for the sole, 1 mm for the articular cartilage, 0.2 mm for the metatarsal cartilage, 1 mm for the bone, and 2 mm for the surrounding soft tissues and performed convergence tests on the discretization of the finite element model, until the calculated stress deviations were less than 5% (see Fig. 1), with the final model consisting of approximately 734,510 nodes and 2,220,835 elements. All bones, cartilages, ligaments and floor were assumed to be linear elastic materials with continuity, complete elasticity, homogeneity, and isotropy. Ideally, the properties should represent the anisotropy of bone. Bone is an orthotropic or anisotropic material, but in most finite element simulations of bone tissue, it is assumed to have an isotropic behavior [14]. We assume bone tissue is an isotropic material mainly for several reasons; first, it is difficult to derive the anisotropic mechanical properties of a given object from conventional CT scans [15]. Second, PENG et al. [16]. performed a finite element comparison study of femurs with two different material properties (i.e., isotropic and anisotropic) and found little difference in the results for the two materials. Third, the loads applied in this study were of physiological magnitude, and with axial loading alone, the use of isotropic bone material properties may not lead to significant differences in stress/strain predictions [17]; approximation may be applicable, therefore, in this study, the material model of bone was simplified to be isotropic material. while we set the soft tissues as nonlinear materials to improve the reliability and accuracy of the experiments to simulate a more realistic quiet tissue state when this volunteer stood, and the floor material was manufactured as a wooden flat plate with a large Young’s modulus wooden flat plate to simulate the ground. The material properties of each component were obtained from the literature [13, 18, 19] (Table 1 lists the material properties of each element).

**Table 1:** Material properties used for various components of the model

Components	Young’s modulus (MPa)	Poisson’s ratio
Ground	17,000	0.1
Soft Tissue	1.15	0.49
Ligaments	260	0
Cartilage	1	0.4
Cortical bone	17,500	0.3
Cancellous bone	300	0.3

### Definition of boundary conditions and loading

In previous studies, for selecting the finite foot element model load size, the value is often taken as half of the subject’s body weight [12, 20, 21]. To improve the realism and accuracy of the finite element model, we performed a bipedal plantar pressure analysis on the volunteer’s foot through the Footwork Pro® (Amcube, France) platform to obtain the average foot pressure on the affected limb and the foot pressure area, and calculated the actual load of the affected foot as 160 N to load the finite element model, considering the influence of the gastrocnemius-fibularis muscle on the accuracy of the finite element model of the stiff clubfoot [22]. In standing, the force generated by the Achilles tendon is approximately 50% of the reaction force on foot [23], and therefore in the finite element model, a force with the direction vertical upward and the size of 80 N was applied to the Achilles tendon; in terms of boundary conditions, the upper surfaces of the tibia, fibula, and soft tissues are entirely fixed, and the friction coefficient between the ground and the soft tissues of the foot is 0.6 [24].

### Validation

**Fig. 2:**
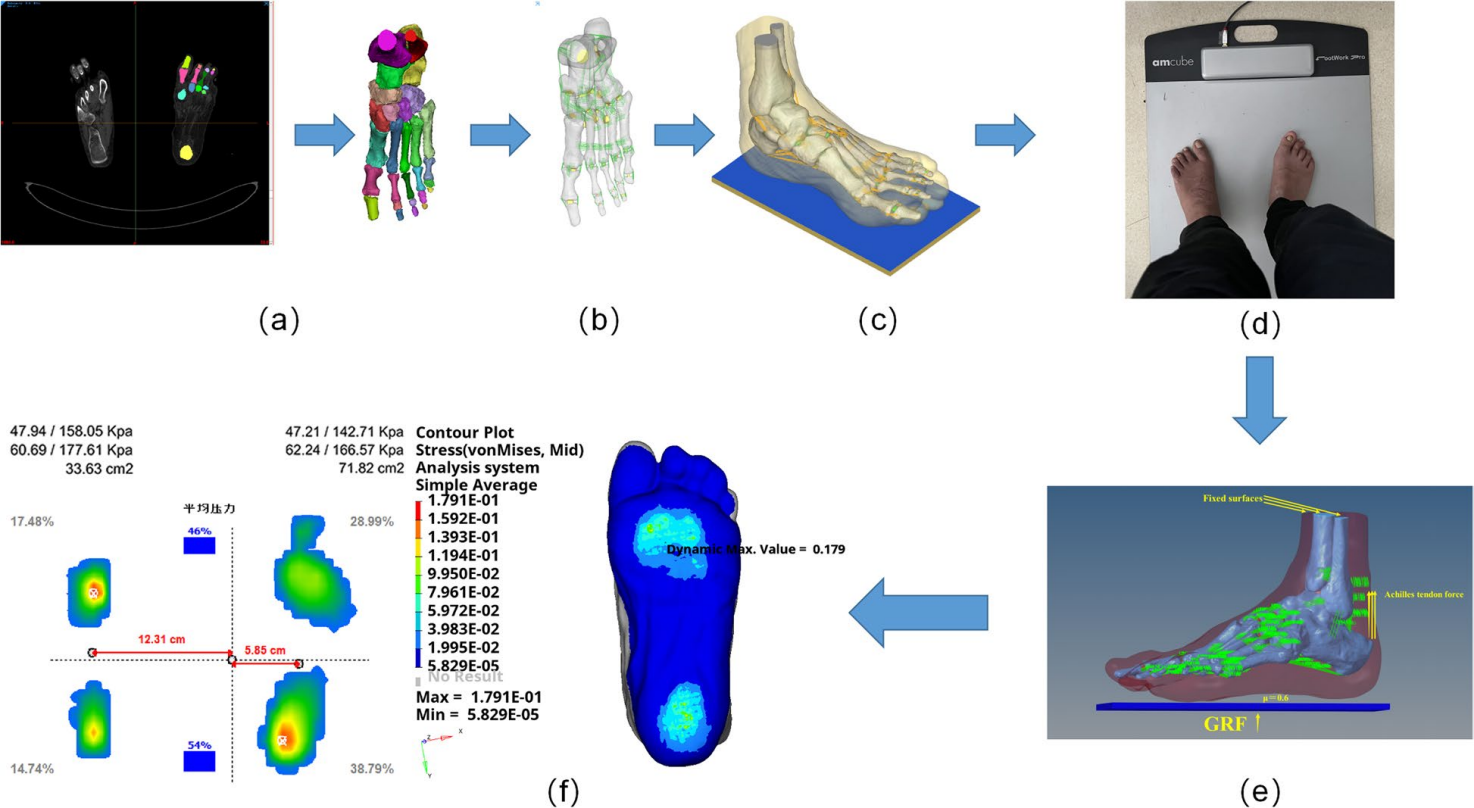
Overview of process steps. **a** Construct 3D skeletal model by segmenting CT images in Mimics software. **b** Construct cartilage and ligament model. **c** Import 3D foot model (bony structures, soft tissues, and ground) into Hypermesh software. **d** Plantar pressure measurement in Footwork Pro® plantar pressure plate system. **e** Apply boundary conditions and loading to the model. **f** Run the model and compare the plantar pressure results from the finite element simulation with the results from the test

To verify the results of the finite element analysis, we performed plantar stress analysis on the volunteer and applied the Footwork Pro® plantar pressure plate system to measure the plantar pressure under standing conditions, obtained the plantar stress distribution cloud map, and loaded the finite element model according to the weight of the affected foot in the plantar pressure results. The results showed that the predicted effects of the FEA and the actual measured plantar peak stress and stress distribution in the standing condition of the volunteer were in good agreement, and the error of the peak von Mises stress was less than 1%. The model validation was effective (see Fig. 2).

### Geometry after TA

**Fig. 3:**
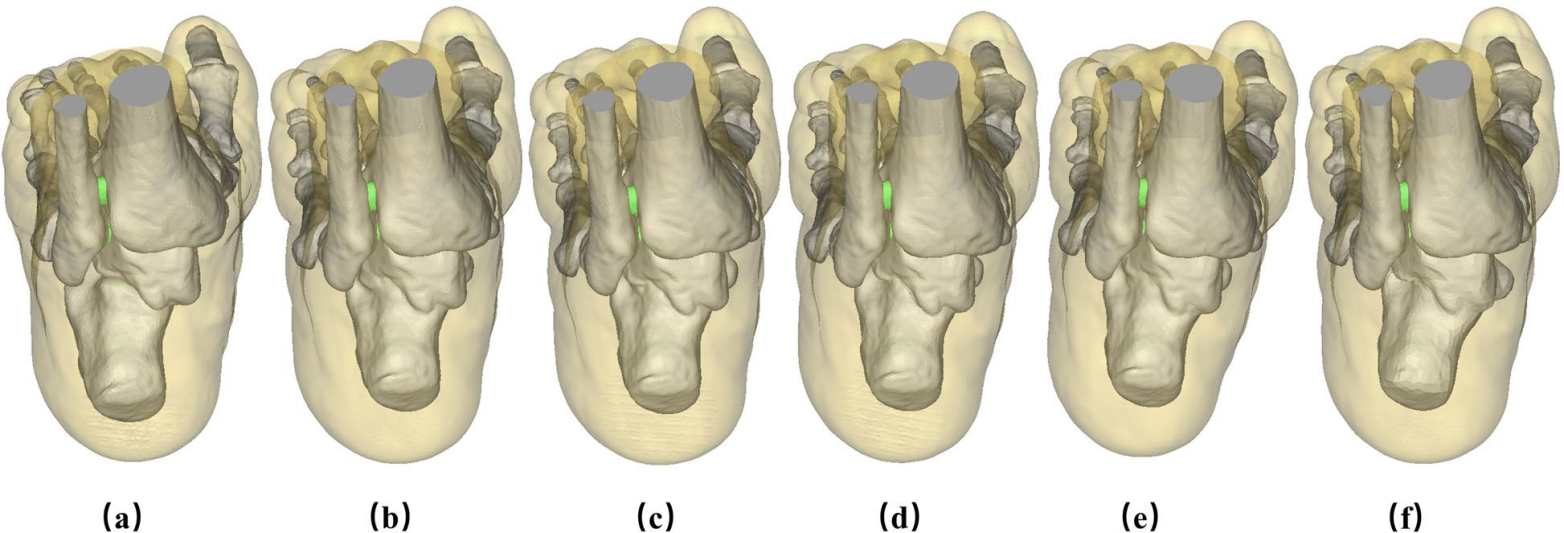
The finite element model of each operating mode before and after operation: (**a**) is the preoperative model, (**b**) is varus “3°”, (**c**) is neutral position “0°”, (**d**) is valgus “3°”, (**e**) is valgus “6°”, (**f**) is valgus “9°”

Based on the validation of the model, we simulated the TA for the stiff clubfoot finite element model. We acted in five different operating modes with correction angles of “3°” for varus, “0°” for neutral, and “3°, 6°, and 9°” for valgus, as well as the preoperative model (see Fig. 3).

### Statistical analysis

Statistical analysis was performed using SPSS version 27.0 software (SPSS Inc., Chicago, IL, USA). One-way ANOVA was used to test the difference in adjacent joint pressure at different correction angles of the hindfoot. *P* < 0.05 was considered to be statistically significant.

## Results

**Fig. 4:**
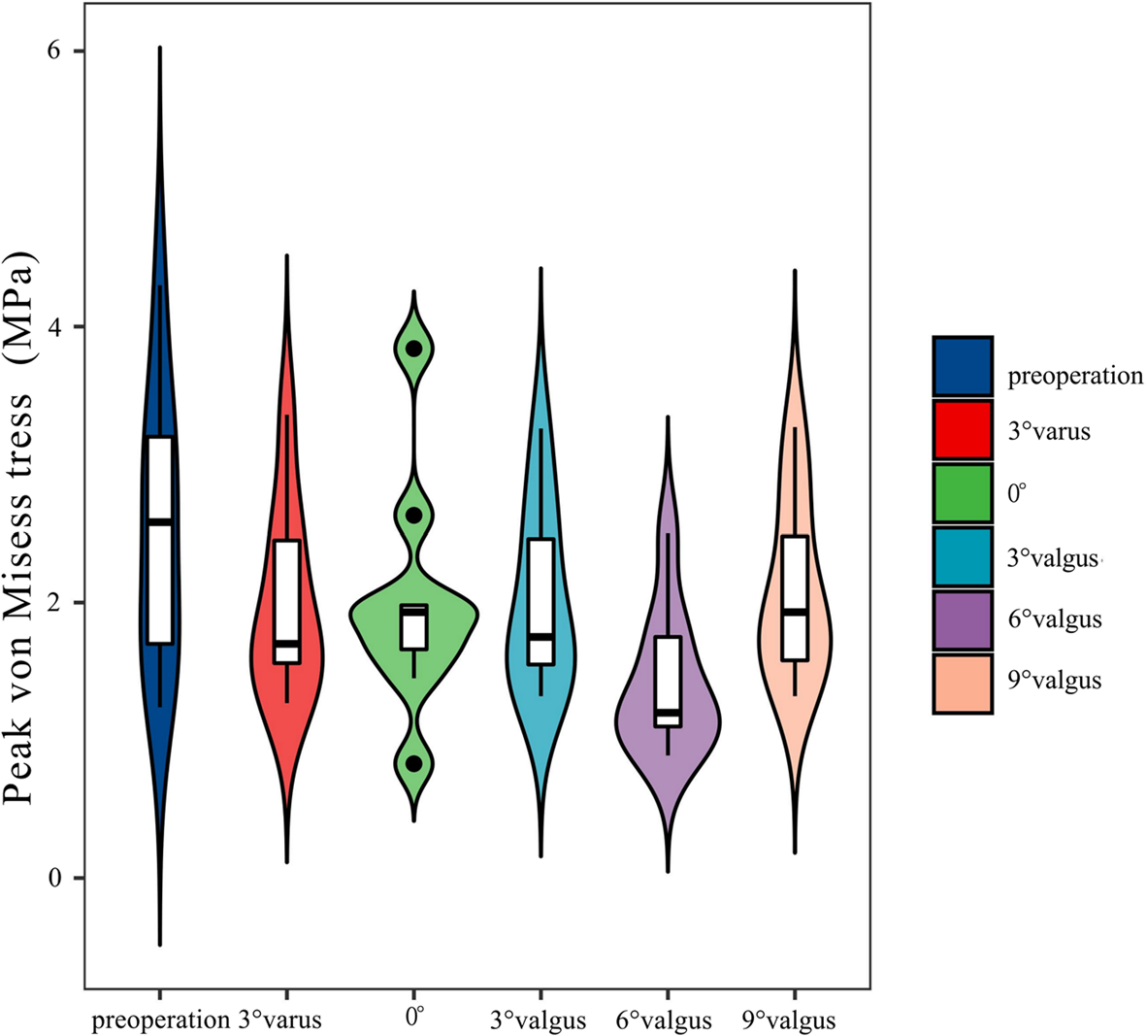
Peak von Mises stress distribution in each operating mode

**Fig. 5:**
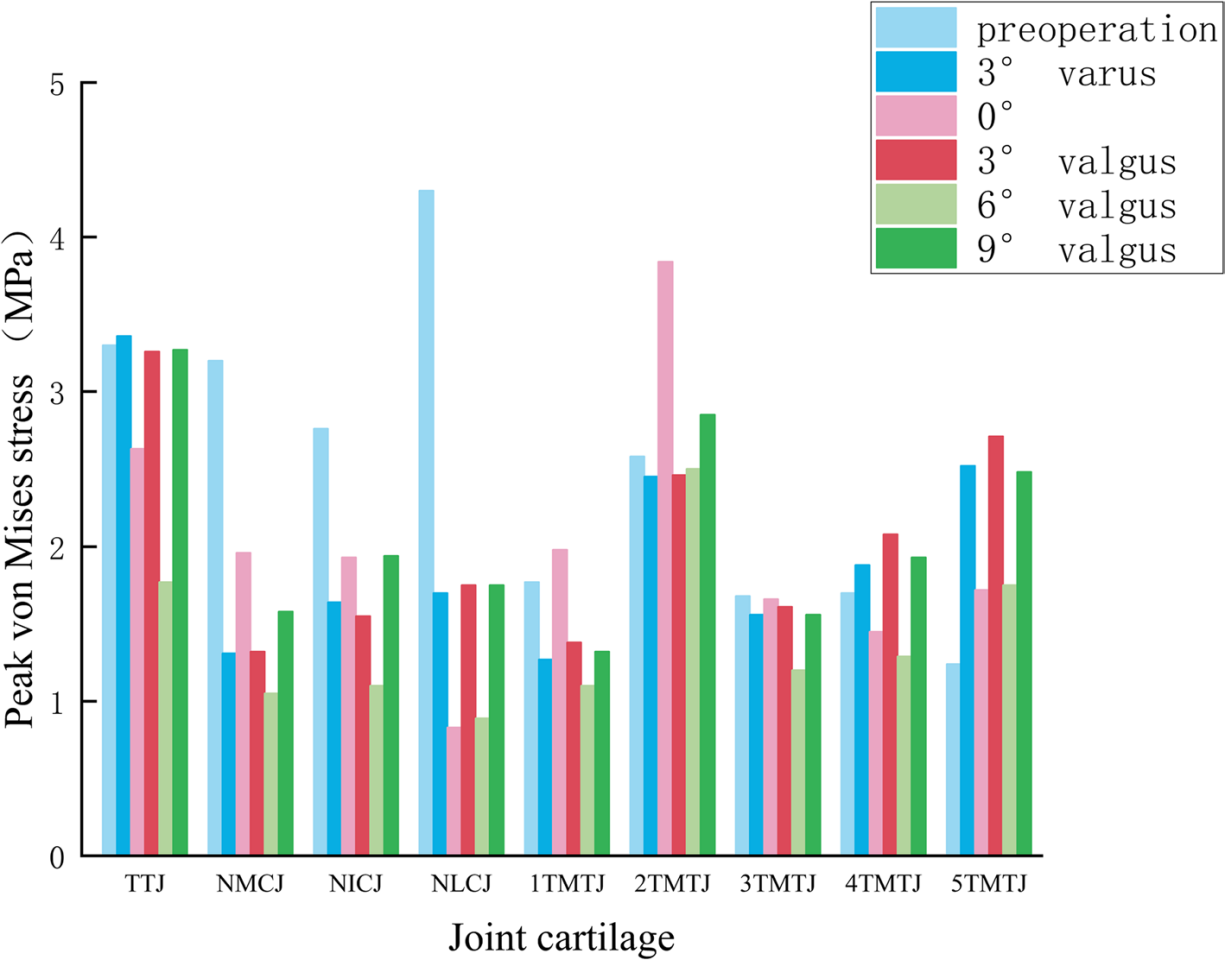
Joint cartilage peak von Mises stress (MPa) in each operating mode

The results indicated that the peak plantar pressure on the affected side was 177.61 KPa, and the peak plantar pressure on the dramatic side was 179.1 KPa obtained by the finite element estimation, with an error of (0.8) %, and the distribution of the peak pressure areas on both sides was in good agreement, with the peak areas being in the second below the metatarsal head, and the finite element model was validated. We obtained the preoperative and postoperative hindfoot angles of varus “3°”, neutral position “0°” and valgus “3°, 6°, 9°” for the TA of the stiff clubfoot by finite element method analysis. “peak von Mises stresses in the articular cartilage of the tibiotalar joint (TTJ), navicular-medial cuneiform joint (NMCJ), navicular-intermediate cuneiform joint (NICJ), navicular-lateral cuneiform joint (NLCJ), first tarsometatarsal joint (1TMTJ), second tarsometatarsal joint (2TMTJ), third tarsometatarsal joint (3TMTJ), fourth tarsometatarsal joint (4TMTJ) and fifth tarsometatarsal joint (5TMTJ). However, through the statistical analysis, we found that the overall stresses in the articular cartilage in the preoperative and postoperative TA with varus “3°”, neutral position “0°” and valgus “3°, 9°” were not statistically significant (*P* > 0.05). The overall stress in the articular cartilage was statistically significant (*P* < 0.05) compared with the postoperative 6° valgus. The overall pressure in the articular cartilage was significantly lower in the 6° valgus condition than in the preoperative condition. The statistical results are shown in Table 2 and Fig. 4. The peak von Mises stress results of each articular cartilage are shown in Table 3 and Fig. 5.

**Table 2:** Statistical results of the overall peak von Mises stress for each operating condition for a two-by-two comparison

Operating mode	Average ± SD (MPa)	Bonferroni method to compare *p* values					
		Preoperation	3°varus	0°	3°valgus	6°valgus	9°valgus
Preoperation	2.5 ± 0.99	—	0.641	0.702	0.725	<0.05	0.823
3°varus	1.97 ± 0.68		—	1	1	0.601	1
0°	2 ± 0.84			—	1	0.537	1
3°valgus	2.01 ± 0.67				—	0.513	1
6°valgus	1.41 ± 0.51					—	0.404
9°valgus	2.08 ± 0.65						—

**Table 3:** Joint cartilage peak von Mises stress (MPa) in each operating mode

Operating mode	Joint cartilage peak von Mises stress (MPa)								
	TTJ	NMCJ	NICJ	NLCJ	1TMTJ	2TMTJ	3TMTJ	4TMTJ	5TMTJ
Preoperation	3.3	3.2	2.76	4.3	1.77	2.58	1.68	1.7	1.24
3°varus	3.36	1.31	1.64	1.7	1.27	2.45	1.56	1.88	2.52
0°	2.63	1.96	1.93	0.83	1.98	3.84	1.66	1.45	1.72
3°valgus	3.26	1.32	1.55	1.75	1.38	2.46	1.61	2.08	2.71
6°valgus	1.77	1.05	1.1	0.89	1.1	2.5	1.2	1.29	1.75
9°valgus	3.27	1.58	1.94	1.75	1.32	2.85	1.56	1.93	2.48
Average ± SD	2.93 ± 0.63	1.74 ± 0.78	1.82 ± 0.55	1.87 ± 1.27	1.47 ± 0.33	2.78 ± 0.54	1.55 ± 0.17	1.72 ± 0.3	2.07 ± 0.58

**Fig. 6:**
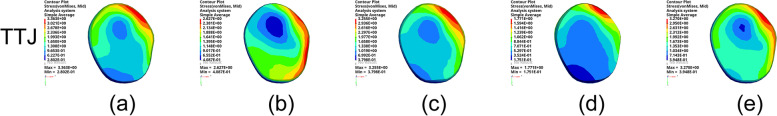
The von Mises stress of TTJ cartilage in each operating mode; (**a**) varus “3°”, (**b**) neutral position “0°”, (**c**) valgus “3°”, (**d**) valgus “6°”, (**e**) valgus “9°”

**Fig. 7:**
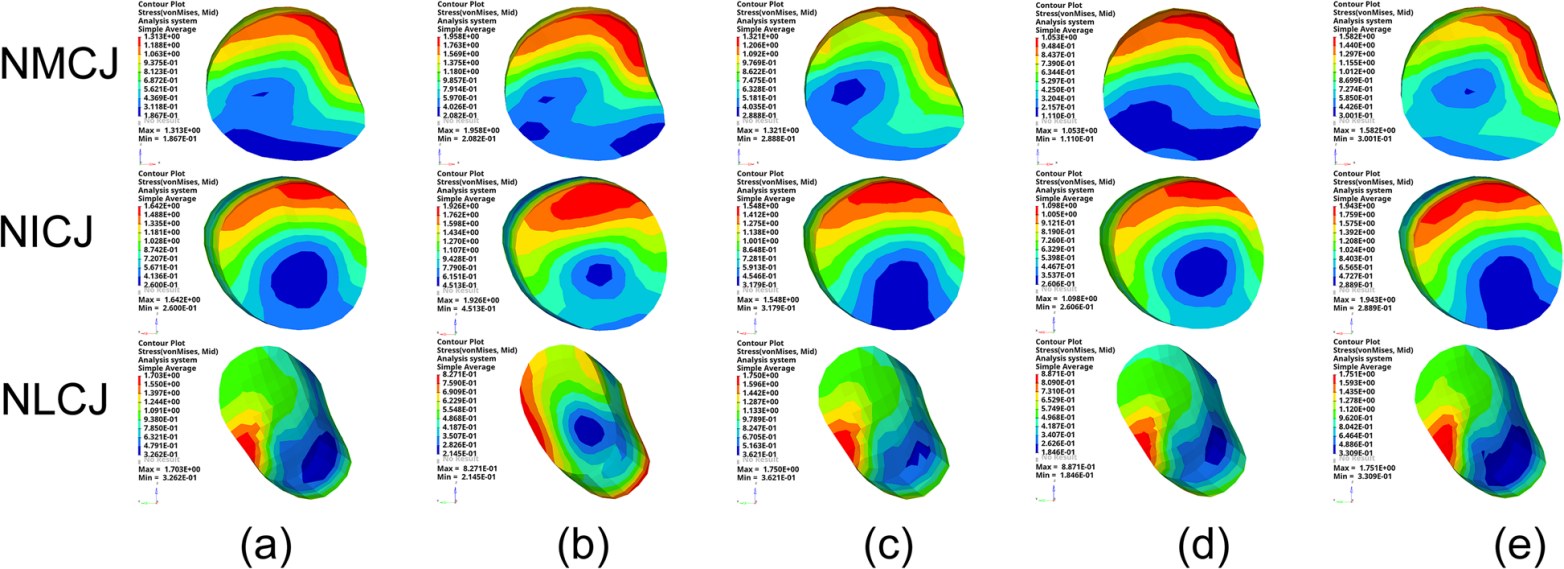
The von Mises stress of NMCJ, NICJ and NLCJ cartilage in each operating mode; (**a**) is varus “3°”, (**b**) is neutral position “0°”, (**c**) is valgus “3°”, (**d**) is valgus “6°”, (**e**) is valgus “9°”

**Fig. 8:**
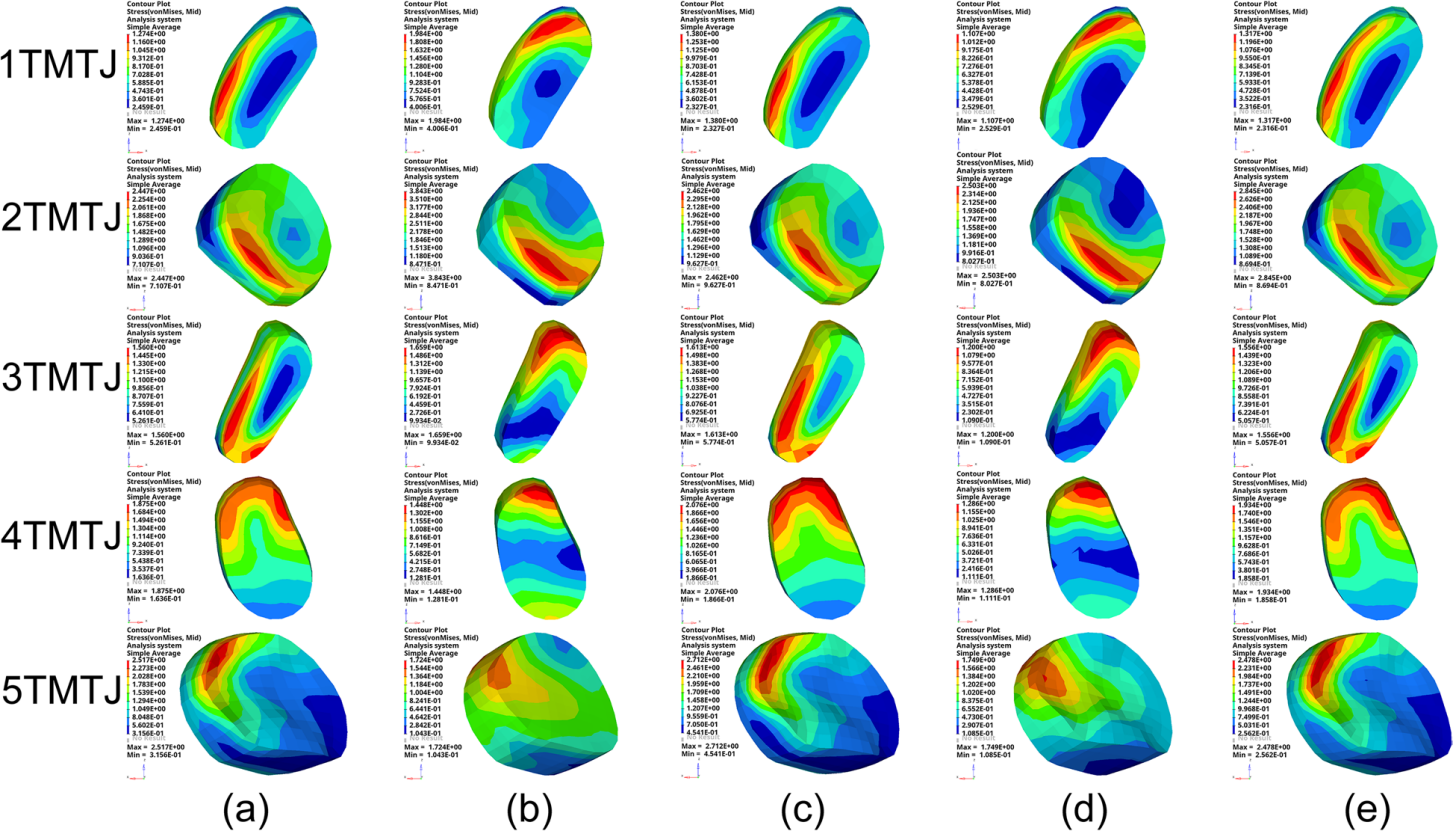
The von Mises stress of 1 TMTJ, 2 TMTJ, 3 TMTJ, 4 TMTJ and 5 TMTJ cartilage in each operating mode; (**a**) is varus “3°”, (**b**) is neutral position “0°”, (**c**) is valgus “3°”, (**d**) is valgus “6°”, (**e**) is valgus “9°”

By comparing the peak von Mises stresses in the TTJ, the maximum stress was 3.36 MPa at “3°” of hindfoot varus, and the minimum pressure was 1.77 MPa at 6° valgus in Fig. 6. The average peak von Mises stress in the three NCJs under each condition was compared. The maximum value of the average peak von Mises stress was 3.42 MPa before TA. The minimum value of the average peak von Mises stress was 1.01 MPa in valgus “6°” in Fig. 7. Comparing the mean peak von Mises stresses in the five TMTJs under each condition, the maximum mean peak von Mises stress occurred in the hindfoot neutral position “0°” with 2.13 MPa, and the minimum mean peak von Mises stress occurred in valgus position “6°”, which was 1.57 MPa in Fig. 8.

## Discussion

Due to the complex anatomy and internal mechanical transfer of the foot and ankle, their biomechanical characteristics are challenging to measure in vivo. Finite element analysis has been used in orthopaedics for more than 20 years. It has been proved that FEA is accurate in orthopaedics, which can guide clinical decision-making and effectively predict the displacement and stress of objects under load [11, 25]. Implementing finite element models to calculate the biomechanical properties inside the foot is feasible and effective [26, 27]. The most significant advantage of 3D finite element models is that they avoid direct invasive experiments on the human body to quantitatively assess the complex biomechanical characteristics of the foot and ankle at a lower cost [28], especially for biomechanical studies of articular cartilage [29, 30]. In the present study, based on previous studies of foot and ankle finite element biomechanics [31, 32], a finite element model of the foot and ankle based on an actual patient with stiff clubfoot was developed and validated by the Footwork Pro® plantar pressure plate system under static standing conditions. The model was validated under static standing conditions with the Footwork Pro® plantar pressure plate system. Unlike previous studies, due to the specificity of this disease, it is often challenging to balance the stresses in both lower limbs when the patient stands [33, 34], and therefore we applied the actual measured total stress data on the affected side during standing to load the finite element model. The model’s validity was verified regarding the magnitude and location of the peak von Mises stresses and the distribution of the overall plantar pressures.

Based on the successful validation of the finite element model, we established a TA model for the five cases of hindfoot varus “3°”, neutral position “0°” and valgus “3°, 6°, 9°”. The postoperative peak von Mises stresses were mainly concentrated in the TTJ cartilage and TMTJ cartilage. In contrast, the preoperative peak von Mises stresses were primarily focused on the NCJ. The postoperative stress distribution was more consistent with the results of Wang et al. [35]. When they performed finite element biomechanical analysis on the standard foot, indicating that TA can improve abnormalities, Yang et al. [36] retrospectively studied 29 adult patients with rigid flatfoot who underwent TA. The results showed that all patients had relief of foot and ankle pain, and 26 received complete follow-ups with an overall satisfaction rate of 84.6% after surgery. The results showed that TA significantly relieved foot pain and corrected force lines, which is consistent with our opinion and supported by good clinical outcomes. Previous studies have suggested that the concentrated distribution of stresses accelerates degenerative changes in articular cartilage [37]. Therefore, the finite element analysis of the TTJ cartilage results in each condition showed that the maximum peak von Mises stress occurred in the hindfoot varus “3°” as the correction angle. Nevertheless, it increased by 0.06 MPa compared to the preoperative condition. The minimum peak von Mises stress appeared in the state of hindfoot valgus “6°”. It decreased by 1.53 MPa or 46% compared with the preoperative condition, indicating that different arthrodesis angles of the hindfoot can significantly affect the stress distribution of the TTJ, and the appropriate arthrodesis angle can dramatically balance the stresses in the TTJ. In our study, we concluded that TA with a “6°” hindfoot valgus as a criterion significantly reduced the stress concentration in the TTJ, thus reducing the risk of TTJ arthritis. Nevertheless, we did not find significant increases in TTJ stress in several other working conditions, probably because of the small number of working conditions. However, we did not see any significant rise in TTJ stress in several different working conditions, probably because of the small number of working condition settings. Therefore, we did not find substantial evidence that TA significantly increases TTJ stress and thus increases the risk of TTJ arthritis, which is consistent with the view of Klerken et al. [4] that rearfoot malalignment after TA does not lead to a higher degree of osteoarthritis in the ankle based on long-term follow-up results.

By studying the peak von Mises stress in the NCJ cartilage, we found that the maximum mean peak von Mises stress in the NCJ cartilage was 3.42 MPa before surgery, and the minimum mean peak von Mises stress was 1.01 MPa at “6°” of valgus, with a mean peak von Mises stress decreased by 70% compared with the preoperative value at “6°” of hindfoot valgus. From the statistical results, it is not only at “6°” of hindfoot valgus that the mean peak von Mises stress decreased significantly in the NCJ. The preoperative peak von Mises stresses were also considerably reduced in the four conditions of varus “3°”, neutral “0°” and valgus “3°, 9°”. Therefore, we can conclude that the TA can significantly change the stress transmission pattern of the midfoot, which can dramatically reduce the load on the NCJ, thus reducing the risk of arthritis in the NCJ. The significant reduction in articular cartilage load can also significantly improve the pain symptoms of the NCJ, while the case of hindfoot valgus “6°” can be reduced considerably. The peak von Mises stress in the NCJ cartilage can be minimized when TA is performed, and the stress distribution is more balanced, which can be used as a reference for the optimal angle of hindfoot arthrodesis when TA is performed.

The main limitation of this study is that the study included only one foot and only studied the finite element model in the unipedal standing condition without checking the biomechanical response of the model when loaded throughout the gait cycle, which may introduce some bias. In future studies, we can include more foot models and load the model throughout the gait cycle to obtain more convincing experimental results. Second, when we studied the cartilage of the TTJ, we did not consider the biomechanics of the cartilage of the inner and outer ankle. Although the contact area of the cartilage of the inner and outer ankle is smaller, it tends to be more prone to degenerative changes than the TTJ; therefore, further studies on the biomechanical changes of the articular cartilage of the inner and outer ankle are necessary for later analyses. In addition, the finite element model of the foot and ankle in this study was not modeled by MRI, which may introduce some bias. In subsequent studies, we can use MRI data to model the finite element model of the foot and ankle to make the results of the survey more accurate and reliable. Finally, we did not consider the effects of different internal fixation methods of joint arthrodesis on the adjacent articular cartilage. The impact of various internal fixation methods of joint arthrodesis on the biomechanics of the model after TA can also be investigated later to obtain a more comprehensive and accurate treatment plan; currently, we have only studied the optimal hindfoot orthopedic angle for TA at the theoretical level, and therefore, all the current findings need further validation by clinical evidence.

## Conclusion

The results of this study showed that different angles of hindfoot correction in TA did not increase the peak von Mises stress in the adjacent joints, which may not lead to the development of arthritis in the adjacent joint, and a hindfoot correction angle of “6°” of valgus significantly reduced the peak von Mises stress in the adjacent joints after TA. Therefore, surgeons should consider the results of this computational exploratory study when selecting a fixation strategy for TA.

## Data Availability

The datasets used and/or analyzed during the current study are available from the corresponding author on reasonable request.

## Abbreviations

ST: talocalcaneal
TN: talonavicular
CC: calcaneocuboid
TA: triple arthrodesis
CT: computed tomography
3D: 3-dimensional
DICOM: Digital Imaging and Communications in Medicine
NURBS: nonuniform rational B-splines
TTJ: tibiotalar joint
NMCJ: navicular-medial cuneiform joint
NICJ: navicular-intermediate cuneiform joint
NLCJ: navicular-lateral cuneiform joint
TMTJ: tarsometatarsal joint
